# Early adolescent outcomes of joint developmental trajectories of problem behavior and IQ in childhood

**DOI:** 10.1007/s00787-018-1155-7

**Published:** 2018-04-16

**Authors:** Eirini Flouri, Efstathios Papachristou, Emily Midouhas, Heather Joshi, George B. Ploubidis, Glyn Lewis

**Affiliations:** 1 Department of Psychology and Human Development, UCL Institute of Education, University College London, 25 Woburn Square, London, WC1H 0AA UK; 2 Department of Social Science, UCL Institute of Education, University College London, London, UK; 3 UCL Division of Psychiatry, University College London, London, UK

**Keywords:** IQ, Problem behavior, Joint trajectories, Externalizing problems, Internalizing problems, Cognitive ability

## Abstract

General cognitive ability (IQ) and problem behavior (externalizing and internalizing problems) are variable and inter-related in children. However, it is unknown how they co-develop in the general child population and how their patterns of co-development may be related to later outcomes. We carried out this study to explore this. Using data from 16,844 Millennium Cohort Study children, we fitted three-parallel-process growth mixture models to identify joint developmental trajectories of internalizing, externalizing and IQ scores at ages 3–11 years. We then examined their associations with age 11 outcomes. We identified a typically developing group (83%) and three atypical groups, all with worse behavior and ability: children with improving behavior and low (but improving in males) ability (6%); children with persistently high levels of problems and low ability (5%); and children with worsening behavior and low ability (6%). Compared to typically developing children, the latter two groups were more likely to show poor decision-making, be bullies or bully victims, engage in antisocial behaviors, skip and dislike school, be unhappy and have low self-esteem. By contrast, children (especially males) in the improver group had outcomes that were similar to, or even better than, those of their typically developing peers. These findings encourage the development of interventions to target children with both cognitive and behavioral difficulties.

**Electronic supplementary material:**

The online version of this article (10.1007/s00787-018-1155-7) contains supplementary material, which is available to authorized users.

## Introduction

Low levels of general cognitive ability (IQ) and high levels of problem behavior (internalizing and externalizing problems) in childhood are independently associated with many adverse outcomes in later life, including psychiatric disorders, all-cause mortality, morbidity, social exclusion and poverty [1–3]. IQ and problem behavior are variable [4, 5, 6, 7–15] and inter-related in children, with evidence for substantial comorbidity between internalizing and externalizing problems [16] and for strong links between IQ and externalizing problems [17]. There is also some evidence for a longitudinal association between various cognitive skills and externalizing problems in the very early years [18], between specific cognitive skills and specific internalizing or externalizing problems at the end of the distribution of either [19, 20] or both [21–23], and between externalizing and internalizing problems [6, 24, 25]. However, the extent to which all three combine in the general child population and over time and whether their combinations are related to later outcomes have not been examined yet.

In this study, we aimed to address this gap by examining the co-development of internalizing problems, externalizing problems and IQ at ages 3–11 years (using three-parallel-process growth mixture modeling) in a general child population cohort. We also tested how the various patterns of their co-development may be associated with behavioral, social and emotional outcomes in early adolescence (age 11 years). These were truancy, antisocial behavior, decision-making, bullying, school engagement, happiness and self-esteem.

## Method

### Sample

The data for this study came from the first five sweeps of the Millennium Cohort Study (MCS), a population-based longitudinal cohort study of children born in the UK over 12 months from 1 September 2000. The children were around 9 months old at Sweep 1, and 3, 5, 7 and 11 years old at Sweeps 2, 3, 4 and 5, respectively. At Sweeps 1, 2, 3, 4 and 5, the number of productive families was 18,522, 15,590, 15,246, 13,857 and 13,287, respectively. The analytic sample of our study included children (singletons and first-born twins or triplets) with valid data on problem behavior and IQ in at least one of Sweeps 2 (when these were first measured in MCS) to 5 (*N* = 16,844; 51% male). Ethical approval was gained from NHS Multi-Centre Ethics Committees, and parents gave informed consent before interviews took place. Figure S1 in the Supplementary Material shows the flowchart of the study design.

### Measures

#### General cognitive ability (IQ) at ages 3, 5, 7 and 11 years

IQ was calculated for each age by using the age-adjusted ability assessments that were available in MCS, as in Flouri et al. [26]. At age 3, there were two cognitive assessments, the Bracken School Readiness Assessment-Revised, which measures children’s ‘readiness’ for formal education by testing their knowledge and understanding of basic concepts [27], and the second edition of the British Ability Scales (BAS) [28] for Naming Vocabulary, which measures expressive language. At age 5, ability was assessed with three scales: BAS Naming Vocabulary, BAS Pattern Construction (measuring spatial problem-solving) and BAS Picture Similarities (measuring non-verbal reasoning). At age 7, it was measured with BAS Pattern Construction, BAS Word Reading (measuring educational knowledge of reading) and the National Foundation for Educational Research Progress in Maths. At age 11, it was measured with BAS Verbal Similarities, which assesses verbal reasoning and verbal knowledge.

When multiple cognitive assessments were available (i.e., at ages 3, 5 and 7), IQ was measured using the scores derived from a principal components analysis of these assessments. Each component score was then transformed into a standardized IQ score with a mean of 100 and a standard deviation of 15 [29]. Multiple well-validated assessments are thought to be able to capture a higher-level intelligence (‘*g*’) factor which is not dependent on the use of specific mental ability tasks [30]. For age 11, when only one measure of ability was available in MCS, we transformed the age-adjusted ability score into a standardized IQ score.

#### Problem behavior (internalizing and externalizing problems) at ages 3, 5, 7 and 11 years

Internalizing and externalizing problems were measured with the parent-reported Strengths and Difficulties Questionnaire (SDQ), a short behavioral screening tool for children aged 2–17 years [31]. The 20 difficulties and symptoms assessed by the SDQ are equally divided in four subscales: emotional symptoms, conduct problems, hyperactivity/inattention and peer problems. In line with the recommended practice for community samples [32], the internalizing problems scale comprised the ten items from the emotional and peer problems subscales, and the externalizing problems scale the ten items from the hyperactivity and conduct problems subscales. Scores on each of these two scales range from 0 to 20, with higher scores indicating more problems or symptoms.

#### Behavioral, social and emotional outcomes at age 11 years

We examined several outcomes at age 11 years, as follows. *Decision*-*making* was measured using the Cambridge Gambling Task (CGT) [33], described in detail in a previous study using it in MCS [34]. The CGT produces six outcomes, *risk taking*, *quality of decision*-*making*, *deliberation time, risk adjustment, delay aversion* and *overall proportion bet. Bullying involvement* was measured with two self-report items: ‘how often do other children hurt you or pick on you on purpose?’ and ‘how often do you hurt or pick on other children on purpose?’ Following much previous research, we identified four mutually exclusive groups of bullying involvement based on children’s answers to these questions: ‘*neutrals’, bullies, victims* and *bully victims*. (Both self-reported items were scored on six-point scales from 1 = most days to 6 = never. Participants who answered ‘never' on both questions were classified as ‘neutrals’, those answering between ‘1’ (most days) and ‘5’ (less often than every few months) on the first and ‘6’ (never) on the second were classified as ‘victims’, those answering between ‘1’ and ‘5’ on the second and ‘6’ on the first were classified as ‘bullies’, and those whose answers on both questions ranged between ‘1’ and ‘5’ were classified as ‘bully victims’.) Single item ‘yes–no’ questions were used to assess *truancy* (‘Have you ever missed school without your parents’ permission?’) and *smoking or drinking* (‘Have you ever had an alcoholic drink, that is, more than a few sips?’ and ‘Have you ever tried a cigarette, even if it was only a single puff?’). *School engagement* was assessed by means of a single item asking the child how much he or she likes school, on a scale from 1 (a lot) to 3 (not at all). A *(un)happiness* measure was derived by summing the MCS questions on how happy the child feels about six different domains, answered on a 7-point Likert scale from 1 (completely happy) to 7 (not at all happy). These domains included school work, appearance, family, friends, the school they go to and life as a whole (Cronbach’s alpha = 0.83). Children in the upper quintile of the summative score were classified as being unhappy. A*ntisocial behavior* was measured by a positive response to engagement in behaviors such as being rude or noisy in public places, stealing, spraying graffiti or vandalizing. Finally, *self*-*esteem* was measured with the five items of the Rosenberg self-esteem scale [35] used in MCS (Cronbach’s alpha = 0.74). Higher scores on the scale (ranging 5–20) indicated lower self-esteem.

#### Early childhood covariates

We controlled for important early predictors of these outcomes as well as IQ and problem behavior in children [34, 36–38]. These were mother-reported socio-demographic, health and lifestyle characteristics at baseline (age 3 years), as follows: child’s *birth weight* (< 2.5 kg or not), *breastfeeding status* and *ethnicity* (white, Indian, Pakistani or Bangladeshi, black, mixed and other), *maternal education* (university degree or not), *maternal smoking status*, *maternal age at child’s birth*, *maternal psychological distress* (assessed using the Kessler K6; Kessler et al. [58]), *family structure* (living with both biological parents or not) and s*ocioeconomic disadvantage*. This was measured (as in Malmberg and Flouri [40]) using a four-item summative index comprising overcrowding (> 1.5 people per room excluding bathroom and kitchen), lack of home ownership, receipt of income support and income poverty (equivalized net family income below 60% of the national median household income).

We also controlled for *household chaos, parent*–*child relationship, parental involvement, quality of emotional support, harsh parental discipline* and *regular bedtimes*, to account for parenting and the home environment at the age 3 baseline. Household chaos was assessed with a 3-item parent report of how calm and organized the atmosphere at the home is (Cronbach’s alpha = 0.68). Parent–child relationship was assessed using the closeness and conflict scales of the Pianta Child–Parent Relationship Scale which comprises 15 items rated on 5-point Likert scales assessing the parent’s perception of their relationship with their child [41]. Parental involvement was assessed with a single item on whether the responding parent reads to their child daily or almost daily. Quality of emotional support was measured using 9 items (Cronbach’s alpha = 0.61) of the short form version of Caldwell and Bradley’s Home Observation for Measurement of the Environment scale [42]. This was a continuous scale ranging 0–9 (with a higher score indicating lower quality) which we dichotomized to 0 vs. ≥ 1 in view of its severe skewness (59% had a score of 0, 28% had a score of 1 and the remaining 13% had a score ≥ 2). Harsh parental discipline was measured using three items (Cronbach’s alpha = 0.66) of the Conflict Tactics Scale [43], asking the parent whether they smack, tell off or shout at the child when he or she misbehaves, with answers ranging from 1 (never) to 5 (daily). Finally, regular bedtimes were assessed by a single item asking whether the child has regular bedtimes. Children whose parent responded ‘sometimes’, ‘almost never’ or ‘never’ were classified as having an irregular bedtime schedule.

#### Analytic strategy

All analyses were stratified by sex in light of the evidence for sex differences in the developmental trajectories of both IQ and problem behavior in childhood [44–47]. In all models, the MCS sampling stratum was controlled to account for the disproportionate stratification of the MCS survey design. All analyses were performed in Stata/SE 14.2 [48] and Mplus 7.4 [49].

To describe the trajectories of internalizing problems, externalizing problems and IQ from ages 3–11 years, we fitted a three-parallel-process growth mixture model (GMM; [50]). In a parallel-process GMM, the growth parameters, i.e., the slope and intercept, are estimated for each of the repeatedly measured variables. Also estimated is a latent class variable, defined by the growth parameters of the parallel processes. We estimated models with 1–6 classes and compared model fit with four commonly used goodness of fit indices [51]: (1) the Bayesian information criterion (BIC); (2) the sample size-adjusted BIC (SSA-BIC); (3) the Akaike information criterion (AIC) and (4) the entropy of each model. Lower BIC, AIC and SSA-BIC values indicate better fit to the data. Entropy ranges from 0 to 1, with higher values indicating that the latent classes are clearly distinguishable (values ≥ 0.80 are considered adequate). Solutions with extremely small classes (≤ 1% of the sample) or with several small classes (< 2% of the sample) were deemed potentially unstable and thus disregarded. The GMM was carried out using the maximum likelihood with robust standard errors estimator, which is robust to non-normality in the data. Full information maximum likelihood (FIML) was used to accommodate missing data in problem behavior and IQ. Under the assumption that the data are missing at random (MAR), FIML can estimate parameters using any available information that is contained in the dataset. (If data are MAR, the missing data generating mechanism is driven by observed/available information in the data.) It is also considered superior to other techniques used to handle missing data in terms of bias and the sampling variability of the parameter estimates produced [52]. To avoid model convergence to local maxima, we increased the number of random starts (250 random sets with 50 optimizations carried out in the final stage). We adjusted for the child’s exact age at the baseline assessment.

We followed a classic three-step analysis whereby, upon selection of the optimal latent class model, the latent class variable was extracted and used as an observed variable for further testing [53]. (One-step approaches whereby the latent class variable is extracted and examined in terms of its associations with covariates and outcomes in the same analytical step are frequently used, but they can be problematic when examining a large number of covariates [54]. In addition, simulations have shown that in models with high entropy values the covariates do not influence class assignment drastically [55].) Upon examination of the distribution of the covariates across the extracted classes, we ran a series of regression models to measure the associations between class membership and the outcomes at age 11. We ran linear, logistic, ordered logistic and multinomial regression models, depending on the scale of measurement of each outcome. These regression models were adjusted for covariates. To handle missing data on them, we used multiple imputation (MI) and not FIML to more flexibly bring into the imputation phase auxiliary variables that maximize the plausibility of the MAR assumption. By including auxiliary variables at both the imputation and analytic stages, MI was performed using the same model specification as the one used in the fully adjusted regression analyses. Thus, we ensured the congeniality between the imputation and the analysis models and increased the likelihood that the MAR assumption was met. We generated 25 imputed datasets using sequential regression models [56].

## Results

Table S1, presented in the Supplementary Material, summarizes the sex-stratified pairwise correlation coefficients and descriptive statistics of internalizing problems, externalizing problems and IQ at children’s ages 3, 5, 7 and 11 years. Internalizing and externalizing problems were positively correlated with each other and negatively with IQ (all *p* values < 0.01). In addition, all three measures showed temporal stability across sexes with Pearson’s correlation coefficients ranging 0.32–0.58 for internalizing problems, 0.47–0.70 for externalizing problems and 0.34–0.61 for IQ (all *p* values < 0.01).

### Parallel-process growth mixture model

In total, data from 16,844 (51% male) children were used in the parallel-process GMM. The fit indices of the competing GMMs are summarized in Table S2 in the Supplementary Material. Starting with the one-class model, stepwise additions of classes resulted in lower BIC, AIC and SSA-BIC values, suggesting better model fit to the data for higher-class solutions. However, the five- and six-class models included several small classes. The four-class solution also had a higher entropy value (0.88) compared to the five- or six-class solutions. Therefore, we considered the four-class solution optimal. In the four-class model, the average latent class (posterior) probabilities for the most likely latent class allocation ranged from 80 to 93% in males and 75 to 94% in females, indicating minimal ambiguity in class assignment.

**Fig. 1:**
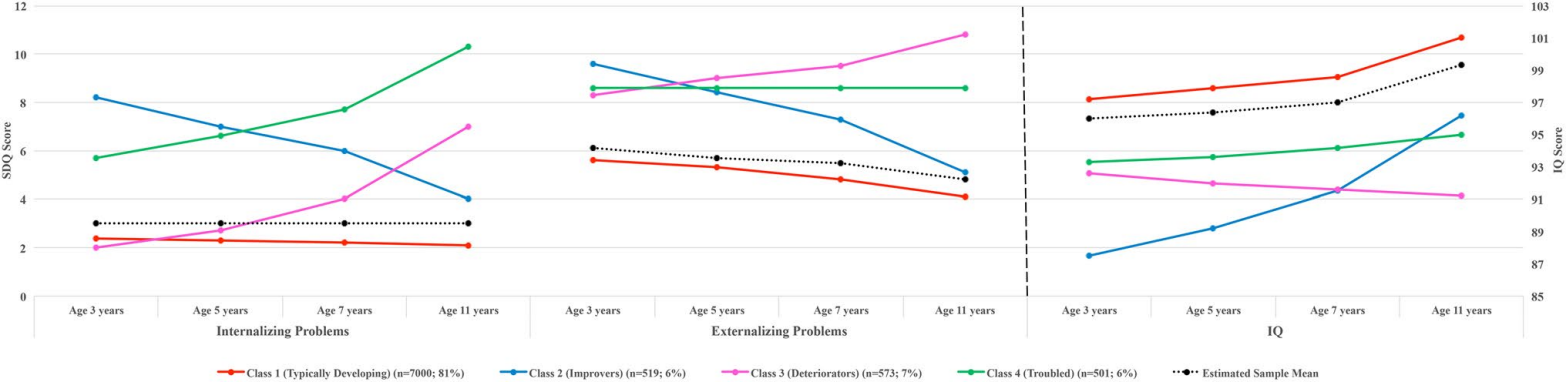
Four-class solution of the three-parallel-process growth mixture model showing the parallel developmental trajectories of SDQ internalizing problems, SDQ externalizing problems and IQ from ages 3–11 years in males

**Fig. 2:**
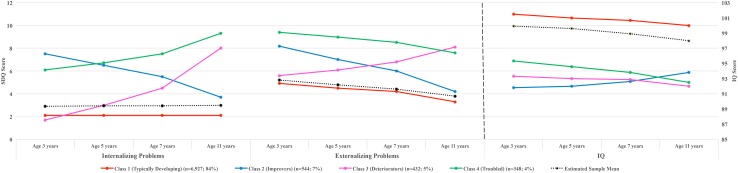
Four-class solution of the three-parallel-process growth mixture model showing the parallel developmental trajectories of SDQ internalizing problems, SDQ externalizing problems and IQ from ages 3–11 years in females

Figures 1 and 2 illustrate the parallel trajectories of problem behavior and IQ across the four classes. Table 1 presents the growth estimates of each trajectory. In both males and females, we identified the following classes. (1) A class comprising *typically developing* children (81% of males and 84% of females) with persistently low levels of internalizing and externalizing problems and with IQ scores above the sample average throughout the study period. (2) A class comprising children (6% of males and 7% of females) with high levels of internalizing and externalizing problems at baseline, which, however, decreased rapidly and significantly (‘*improvers*’). The IQ of males in this class was approximately one SD below the total sample mean at baseline (intercept = 87.48, *p* < 0.001), but was increasing with an average linear rate of 2.5 points at each assessment (slope = 2.45, *p* < 0.001). The IQ of their female counterparts was also low at baseline (intercept = 91.79, *p* < 0.001), yet the mean slope value did not reach statistical significance (slope = 0.54, *p* > 0.05), suggesting that females’ IQ scores remained stable in this group. (3) A class comprising children with positive and highly significant slope values for both problem domains (*p* values < 0.01), suggesting steadily increasing internalizing and externalizing problem scores during the 8-year study period (‘*deteriorators*’). The IQ of both males and females in this class was persistently low (intercepts = 92.62 and 93.32, respectively; *p* values of slopes > 0.05) across assessments. (4) A class comprising children with low levels of IQ and high levels of internalizing and externalizing problem scores (‘*troubled*’). Internalizing problems in males in this group were increasing at each assessment (slope = 1.13, *p* < 0.01), while females showed very slowly decreasing levels of externalizing problems (slope = − 0.56, *p* < 0.01). Both sexes had IQ scores consistently below the sample mean across assessments. (Note: the average IQ trajectory presented in the figures is the mean estimated trajectory conditional on the behavior trajectories. As a result, it was unlikely that it would overlap perfectly with the mean observed IQ trajectory computed independently of the co-developing behavior trajectories.)

**Table 1:** Growth estimates [intercept (*I*) and slope (*S*) and associated standard error (SE)] of internalizing problems, externalizing problems and IQ, stratified by class membership and sex

	Internalizing problems		Externalizing problems		IQ	
	*I* (SE)	*S* (SE)	*I* (SE)	*S* (SE)	*I* (SE)	*S* (SE)
Males						
Typically developing	**2.33 (0.06)**	**− 0.06 (0.01)**	**5.71 (0.09)**	**− 0.41 (0.02)**	**97.36 (0.49)**	**0.87 (0.11)**
Improvers	**8.20 (0.29)**	**− 1.10 (0.09)**	**9.57 (0.34)**	**− 1.18 (0.11)**	**87.48 (1.51)**	**2.45 (0.39)**
Deteriorators	**2.03 (0.18)**	**1.39 (0.11)**	**8.24 (0.32)**	**0.72 (0.09)**	**92.62 (1.17)**	**− **0.34 (0.41)
Troubled	**5.79 (0.37)**	**1.13 (0.16)**	**8.55 (0.33)**	**− **0.01 (0.11)	**93.38 (1.46)**	0.45 (0.43)
Females						
Typically developing	**2.14 (0.06)**	0.02 (0.02)	**4.91 (0.09)**	**− 0.44 (0.02)**	**101.57 (0.56)**	**− 0.37 (0.11)**
Improvers	**7.52 (0.27)**	**− 0.93 (0.20)**	**8.16 (0.42)**	**− 1.04 (0.11)**	**91.79 (1.33)**	0.54 (0.48)
Deteriorators	**1.74 (0.40)**	**1.80 (0.16)**	**5.74 (0.81)**	**0.65 (0.21)**	**93.32 (1.29)**	**− **0.33 (0.34)
Troubled	**6.09 (1.15)**	0.85 (0.61)	**9.37 (0.70)**	**− 0.56 (0.21)**	**95.35 (1.94)**	**− **0.72 (0.48)

Bold indicates *p* values < 0.01

Visual inspection of the data in the figures suggested that the trajectories were linear. We tested this formally by fitting a four-class parallel-process GMM including a quadratic growth parameter. The entropy of the GMM with the quadratic growth parameter was comparable (0.87) to the one without. We also tested the concordance rate of class allocation between the two competing GMMs by a kappa agreement coefficient. The results showed a concordance rate of 92 and 93% for class allocation in males and females, respectively, suggesting that the two GMMs yield almost identical classifications.

Finally, we tested for non-response bias in the analytic sample by comparing those with complete data on all outcome measures across all time points (*N* = 8201) with those with some missingness in the outcomes (*N* = 8643) on key baseline (age 3) characteristics. As expected, there was bias. Children with complete data in all outcome measures had higher IQ scores (*M* = 102.72, SE = 0.16) compared to those without (*M* = 96.05, SE = 0.21). In addition, they scored, on average, 0.53 and 0.86 points lower on the internalizing and externalizing problem scales, respectively (*p* < 0.001). They were also more likely to live with both biological parents (84% vs. 75%, *p* < 0.001), had a higher mean birth weight (mean difference was 0.08 kg, *p* < 0.01) and experienced less socioeconomic disadvantage (*p* < 0.001).

### Outcomes in early adolescence

We fitted regression models on the sample of 13,058 (50% male) children with available data in at least one of the outcomes considered at age 11 years. Those excluded were predominantly male (*p* = 0.001) and had both higher levels of internalizing and externalizing problems and lower levels of IQ scores at all assessments (all *p* values ≤ 0.001). However, the proportion of children with valid data on the outcomes was similarly distributed across classes, ranging from 75 to 93%.

Tables 2 and 3 summarize the characteristics of the sample by class membership and sex. Overall, at age 3 typically developing females and males experienced less socioeconomic disadvantage and more parental involvement, had older, more educated and less distressed mothers, enjoyed better child–parent relationships, lived in less chaotic homes, were less harshly disciplined and were more likely to live with both biological parents and have regular bedtimes, compared to the remaining three classes (all *p* values < 0.01). As the tables show, there were also many differences in these characteristics between the three smaller classes (for example, we note a striking over-representation of ethnic minority children among the ‘improvers’). Therefore, all these variables were adjusted for as covariates in the regression models. The variance inflation factor for these covariates ranged from 1.01 to 1.87, suggesting that they were not highly collinear.

**Table 2:** Distribution across classes of the covariates at baseline (age 3), **males** (unweighted data)

Continuous variables	Typically developing (C1)	Improvers (C2)	Deteriorators (C3)	Troubled (C4)	Significant pairwise comparisons (Bonferroni)
	*M* ± SD	*M* ± SD	*M* ± SD	*M* ± SD	
Socioeconomic disadvantage	0.76 (1.09)	1.58 (1.30)	1.28 (1.30)	1.23 (1.23)	C1 < C2; C1 < C3; C1 < C4; C2 > C3; C2 > C4
Maternal age at birth	28.73 (5.86)	26.89 (6.32)	27.20 (5.97)	27.14 (5.94)	C1 > C2; C1 > C3; C1 > C4
Low household chaos	11.05 (2.13)	10.38 (2.44)	10.23 (2.25)	10.20 (2.52)	C1 > C2; C1 > C3; C1 > C4
Parent–child conflict	16.68 (5.57)	22.57 (6.83)	18.95 (5.89)	21.41 (6.64)	C1 < C2; C1 < C3; C1 < C4; C2 > C3; C2 > C4; C3 < C4
Parent–child closeness	33.55 (2.35)	31.45 (4.16)	32.84 (2.84)	32.45 (3.37)	C1 > C2; C1 > C3; C1 > C4; C2 < C3; C2 < C4
Maternal psychological distress	2.93 (3.42)	6.18 (5.11)	4.19 (4.47)	5.54 (4.79)	C1 < C2; C1 < C3; C1 < C4; C2 > C3; C3 < C4
Harsh parental discipline	9.47 (2.35)	9.79 (2.60)	10.11 (2.48)	9.99 (2.52)	C1 < C3; C1 < C4

Categorical variables	Typically developing (C1)	Improvers (C2)	Deteriorators (C3)	Troubled (C4)	Chi square test *p* value
	*N* (%)	*N* (%)	*N* (%)	*N* (%)	
Ethnicity					
White	5900 (84%)	316 (61%)	511 (89%)	414 (83%)	< 0.001
Mixed	202 (3%)	19 (4%)	12 (2%)	16 (3%)	
Indian	167 (2%)	35 (7%)	9 (2%)	12 (2%)	
Pakistani or Bangladeshi	380 (5%)	108 (21%)	22 (4%)	35 (7%)	
Black	260 (4%)	26 (5%)	15 (3%)	15 (3%)	
Other	87 (1%)	15 (3%)	4 (1%)	9 (2%)	
Mother has university degree	1193 (18%)	49 (10%)	40 (7%)	52 (11%)	< 0.001
Lives with both biological parents	5166 (82%)	326 (69%)	347 (69%)	310 (70%)	< 0.001
Not breastfed	2004 (30%)	182 (38%)	215 (39%)	165 (34%)	< 0.001
Low quality of emotional support	2219 (40%)	208 (58%)	235 (52%)	177 (47%)	< 0.001
Parent reads to child daily	3676 (59%)	191 (41%)	246 (49%)	211 (48%)	< 0.001
Irregular bedtimes	1196 (19%)	162 (35%)	135 (27%)	145 (33%)	< 0.001
Low birth weight	383 (6%)	59 (12%)	43 (8%)	38 (8%)	< 0.001
Mother smokes	1756 (28%)	167 (36%)	215 (43%)	179 (40%)	< 0.001

Results of chi square tests for categorical variables and one-way ANOVA for continuous variables

**Table 3:** Distribution across classes of the covariates at baseline (age 3), **females** (unweighted data)

Continuous variables	Typically developing (C1)	Improvers (C2)	Deteriorators (C3)	Troubled (C4)	Significant pairwise comparisons (Bonferroni)
	*M* ± SD	*M* ± SD	*M* ± SD	*M* ± SD	
Socioeconomic disadvantage	0.79 (1.11)	1.52 (1.30)	1.12 (1.24)	1.54 (1.41)	C1 < C2; C1 < C3; C1 < C4; C2 > C3; C3 < C4
Maternal age at birth	28.79 (5.87)	26.36 (6.29)	27.85 (6.00)	26.88 (6.01)	C1 > C2; C1 > C3; C1 > C4; C2 < C3
Low household chaos	11.23 (2.09)	10.42 (2.36)	10.59 (2.20)	9.90 (2.56)	C1 > C2; C1 > C3; C1 > C4; C2 > C4; C3 > C4
Parent–child conflict	16.34 (5.55)	20.98 (6.46)	17.60 (5.58)	22.43 (6.51)	C1 < C2; C1 < C3; C1 < C4; C2 > C3; C2 < C4; C3 < C4
Parent–child closeness	33.84 (1.99)	32.34 (4.05)	33.70 (2.19)	32.67 (3.28)	C1 > C2; C1 > C4; C2 < C3; C3 > C4
Maternal psychological distress	2.82 (3.26)	6.07 (5.35)	4.33 (4.50)	5.99 (5.26)	C1 < C2; C1 < C3; C1 < C4; C2 > C3; C3 < C4
Harsh parental discipline	9.04 (2.34)	9.22 (2.65)	9.52 (2.48)	9.97 (2.47)	C1 < C3; C1 < C4; C2 < C4

Categorical variables	Typically developing (C1)	Improvers (C2)	Deteriorators (C3)	Troubled (C4)	Chi square test *p* value
	*N* (%)	*N* (%)	*N* (%)	*N* (%)	
Ethnicity					
White	5836 (84%)	342 (63%	374 (87%)	285 (82%)	< 0.001
Mixed	209 (3%)	15 (3%)	16 (4%)	15 (4%)	
Indian	170 (2%)	23 (4%)	6 (1%)	7 (2%)	
Pakistani or Bangladeshi	395 (6%)	119 (22%)	21 (5%)	24 (7%)	
Black	231 (3%)	26 (5%)	9 (2%)	11 (3%)	
Other	83 (1%)	19 (3%)	6 (1%)	6 (2%)	
Mother has university degree	1242 (19%)	29 (6%)	41 (10%)	29 (9%)	< 0.001
Lives with both biological parents	5119 (82%)	362 (74%)	284 (75%)	189 (59%)	< 0.001
Not breastfed	2072 (31%)	207 (41%)	152 (37%)	136 (42%)	< 0.001
Low quality of emotional support	2041 (37%)	206 (53%)	147 (45%)	135 (49%)	< 0.001
Parent reads to child daily	3783 (61%)	216 (44%)	203 (54%)	162 (51%)	< 0.001
Irregular bedtimes	1242 (20%)	176 (36%)	85 (22%)	116 (36%)	< 0.001
Low birth weight	455 (7%)	76 (15%)	52 (13%)	27 (8%)	< 0.001
Mother smokes	1700 (27%)	172 (35%)	141 (37%)	145 (46%)	< 0.001

Results of chi square tests for categorical variables and one-way ANOVA for continuous variables

Tables S3–S8, presented in the Supplementary Material, summarize the results of the crude and adjusted regression models examining the relations between class membership and the age 11 outcomes. Compared to typically developing males, *deteriorators* were more likely to be bullies (OR = 1.80, 95% CI 1.04–3.11), bully victims (OR = 2.98, 95% CI 2.19–4.06) or victims (OR = 2.16, 95% CI 1.56–2.99), to skip school (OR = 2.83, 95% CI 1.95–2.11), to dislike school (OR = 1.75, 95% CI 1.34–2.27), to be unhappy (OR = 2.97, 95% CI 2.25–3.92), to engage in antisocial behaviors (OR = 1.94, 95% CI 1.54–2.55), to smoke or drink (OR = 1.59, 95% CI 1.15–2.20) and to report lower self-esteem (*b* = 0.62, SE = 0.16). *Troubled* males showed less delay aversion, lower quality of decision-making and poorer risk adjustment, compared to typically developing males. They were also more likely to be bully victims (OR = 2.16, 95% CI 1.57–2.98) or victims (OR = 2.38, 95% CI 1.68–3.37), to dislike school (OR = 1.54, 95% CI 1.16–2.05), to be unhappy (OR = 2.56, 95% CI 1.87–3.50), to engage in antisocial behaviors (OR = 1.52, 95% CI 1.14–2.02) and to have lower self-esteem (*b* = 0.71, SE = 0.17). The outcomes of the *improver* males, generally not different from those of their typically developing peers, were in fact better for antisocial behaviors (OR = 0.66, 95% CI 0.48–0.91).

Among females, those classified as *deteriorators* differed significantly from their typically developing peers in all decision-making outcomes considered. Specifically, they had higher scores in risk taking, delay aversion, deliberation time and overall proportion bet, and significantly lower scores in quality of decision-making and risk adjustment (all *p* values < 0.05). In addition, they were more likely to be bully victims (OR = 2.34, 95% CI 1.62–3.37) or victims (OR = 2.74, 95% CI 1.99–3.78), to skip school (OR = 2.22, 95% CI 1.17–4.22), to dislike school (OR = 1.92, 95% CI 1.48–2.50), to be unhappy (OR = 2.76, 95% CI 2.12–3.59), to engage in antisocial behaviors (OR = 1.95, 95% CI 1.44–2.65) and to have lower self-esteem (*b* = 0.71, SE = 0.17). *Troubled* females scored higher on delay aversion and lower on risk adjustment, compared to typically developing females. Moreover, they were significantly more likely to be bully victims (OR = 1.76, 95% CI 1.11–2.79) or victims (OR = 2.70, 95% CI 1.90–3.84), to skip school (OR = 4.15, 95% CI 2.14–8.04), to dislike school (OR = 1.51, 95% CI 1.08–2.10), to be unhappy (OR = 1.90, 95% CI 1.30–2.78) and to have lower self-esteem (*b* = 0.54, SE = 0.20). Finally, females classified as *improvers* were more likely to skip school (OR = 2.35, 95% CI 1.24–4.43), to be unhappy (OR = 1.47, 95% CI 1.06–2.06) and to have lower self-esteem (*b* = 0.44, SE = 0.17), compared to their typically developing peers.

### Children with intellectual disability (ID)

At age 3, none of the children participating in MCS had IQ scores below 55. Thus, there were no children with moderate or severe ID at baseline in our sample. [DSM-IV classifies ID (mental retardation) into four stages based on severity: mild (IQ score of 50–55 to approximately 70), moderate (IQ score of 30–35 to 50–55), severe (IQ score of 20–25 to 35–40), and profound (IQ score of less than 20–25).] Our analytic sample likely excludes children with moderate or severe ID, as it was conditional on cognitive assessment data being available. However, for few MCS children, interviewers did not administer the cognitive assessments. This was the case if the child ‘has a learning disability/serious behavioral problem (e.g., severe ADHD, autism) which prevents them from carrying out the assessments’, ‘is unable to respond in the required manner for each assessment, e.g., reading, writing, manipulating objects’, ‘is not able to speak or understand English (or Welsh if applicable)’ or if consent and co-operation were not forthcoming (see also [57]).

Therefore, our study could only describe the trajectories of non-ID children or children with mild ID. As expected, the lowest proportion of children with mild ID was found in the class of typically developing children (2.5% of boys and 1.3% of girls in this class). However, the majority of children with mild ID were in the “improver” class—and not the “troubled” or “deteriorator” classes—for both males and females (12.4% of boys and 7.5% of girls in this class had IQ scores below 70 at baseline). This finding suggests that low cognitive ability in early childhood does not condemn children to adverse outcomes in early adolescence.

## Discussion

In this study, we examined the unfolding and consequences of the joint development of IQ and problem behavior at ages 3–11 years in the general population. We identified four distinct patterns of this co-development. The *typically developing* children, the majority of the sample, had low levels of problem behavior and normal IQ. At the other extreme, the *troubled* children (6% of males and 4% of females) were those with persistently low IQ and high levels of internalizing and externalizing problems. There were two groups showing significant longitudinal change in problem behavior. The first included 7% of males and 5% of females (*deteriorators*) with low IQ and steadily increasing internalizing and externalizing problems. Like the *troubled* group, these children were at risk of multiple adverse outcomes in early adolescence. The second included 6% of males and 7% of females (*improvers*) with initially high levels of internalizing and externalizing problems that decreased steadily during the study period, reaching the normal range by age 11 years, and low (but rapidly increasing in males) IQ scores. The males in this group had outcomes similar to, or even better than, those of their typically developing peers. The females were as likely as their typically developing peers to engage in risky or antisocial behaviors, although they were, in general, unhappier.

The patterns of the longitudinal association between internalizing and externalizing problems we found in this study resemble previous classifications of developmental patterns of internalizing and externalizing problems in childhood and adolescence [6, 11, 58]. These show that emotional and behavioral problems are largely stable throughout childhood for the majority of children, but also identify subgroups of children with increasing or decreasing symptoms. The findings of our study suggest that this typology holds in MCS too, with the majority of our sample showing either persistently low or persistently high symptom levels throughout childhood. Both in these groups and in the two groups with changing symptoms over time, internalizing and externalizing problems appeared to be positively inter-related and to develop largely in parallel, in line with previous research suggesting high levels of comorbid externalizing and internalizing problems in childhood [6, 59].

Our study also showed an inverse relation between problem behavior and IQ, in line with much evidence for low cognitive skills among children with emotional and behavioral difficulties [20, 60–62]. However, it also showed that the development of problem behavior and IQ was symmetric only for some (especially male) children. Importantly, the small minority of at-risk males with parallel improvement of ability and behavior had outcomes similar to those of their typically developing peers.

### Strengths and limitations

This was the first study to examine the parallel (joint) developmental trajectories of problem behavior and IQ in the general child population, and to explore their links with a range of later social, emotional and behavioral outcomes. Its additional strengths are its large sample size and the prospectively collected data from toddlerhood until the end of primary school. However, the study has some important limitations too. First, it did not explicitly control for parental IQ or paternal mental health. Arguably, we accounted for much of the influence of both, however, by adjusting for maternal mental health (associated with paternal mental health) and education (linked to both IQ and, due to assortative mating, paternal education). Second, it did not control for changes in parental mental health or parenting behavior over the observation period, or for changes in family circumstances after age 3. Third, it could not account for genetic influences on children’s trajectories. Fourth, it was based on a sample that despite its provenance ended up being selective. For example, there was evidence for non-response bias. An additional source of bias in MCS is attrition due to dropout, death or other factors. Attrition rates in each sweep have been consistently higher for families in areas with high proportions of ethnic minorities or disadvantage compared with families in more advantaged areas [63]. However, we attempted to minimize the effect of such bias by using state-of-the-art methods of handling missing data. Fifth, it was unable to allow for the effects of clinical diagnoses (and their treatment or lack thereof), which may be particularly significant for the three atypical groups we identified [64]. Finally, some of the analyses may have been underpowered (for example, the ones predicting truancy due to the low endorsement of positive responses).

## Conclusions

This study explored how IQ and problem behavior (internalizing and externalizing problems) in the general child population are related across development (ages 3–11 years). In general, it showed evidence for an asymmetric longitudinal relationship between IQ and problem behavior in most children. The small minority of at-risk children who showed parallel improvement of behavior and cognitive ability had outcomes that were identical to, or even better than, those of their typically developing peers. Their outcomes were also significantly better than those of at-risk children with improvements only in behavior. Together, these findings suggest that interventions to improve behavior and cognitive skills in children at risk may be particularly effective in improving their later outcomes and life chances. Future studies should test this formally. They could also test if differences in outcomes remain when outcomes are located further in time, and what factors might explain how behavior and cognitive ability co-develop in children in the first place.

## Electronic supplementary material

Below is the link to the electronic supplementary material.

Supplementary material 1 (DOCX 91 kb)
