# Supplementary material for: Early adolescent outcomes of joint developmental trajectories of problem behavior and IQ in childhood
Source: Eur Child Adolesc Psychiatry. 2018 Apr 16;27(12):1595–605. doi: 10.1007/s00787-018-1155-7 (PMC6245124; doi:10.1007/s00787-018-1155-7)
Supplement: Supplementary file 1 — Supplementary material 1 (DOCX 91 kb) [file 787_2018_1155_MOESM1_ESM.docx]

**Supplementary Material**

Figure S1 shows the flow chart of the study design. Overall, 17,356 children participated in at least one of Sweeps 2 (age 3) to 5 (age 11) of MCS. Of those, 2.95% (512/17356) had insufficient data and were excluded from the growth mixture modelling (GMM) analysis; 49.80% (8643/17356) had incomplete data but were included in the analysis; and finally, 47.25% (8201/17356) had complete data in both behavior and cognitive ability across assessments.

Table S1 summarizes the pairwise correlations and descriptive statistics of internalizing problems, externalizing problems and IQ at children’s ages 3, 5, 7 and 11 years.

Table S2 presents the fit indices of 4- to 6- class solutions of three-parallel-process growth mixture models (GMMs) examining the joint developmental trajectories of internalizing problems, externalizing problems and IQ from ages 3 to 11 years.

Tables S3-S8 summarize the crude and adjusted estimates of regression models examining the relationships between class membership and the following outcomes at age 11 years: decision-making (measured with the CGT), bullying involvement, antisocial behavior, truancy, smoking/drinking, school liking, happiness and self-esteem.

**Figure S1.** Flow chart of the study design

| **Table S1.** Pairwise Pearson’s correlations and descriptive statistics of the key observed study variables stratified by sex (unweighted data) | | | | | | | | | | | | |
| --- | --- | --- | --- | --- | --- | --- | --- | --- | --- | --- | --- | --- |
|  | 1 | 2 | 3 | 4 | 5 | 6 | 7 | 8 | 9 | 10 | 11 | 12 |
| 1. Internalizing problems, age 3 | - | 0.49** | 0.41** | 0.32** | 0.38** | 0.27** | 0.24** | 0.22** | -0.22** | -0.17** | -0.15** | -0.15** |
| 1. Internalizing problems, age 5 | 0.48** | - | 0.58** | 0.46** | 0.30** | 0.40** | 0.32** | 0.28** | -0.20** | -0.18** | -0.18** | -0.14** |
| 1. Internalizing problems, age 7 | 0.43** | 0.57** | - | 0.56** | 0.30** | 0.37** | 0.47** | 0.35** | -0.20** | -0.19** | -0.22** | -0.17** |
| 1. Internalizing problems, age 11 | 0.33** | 0.42** | 0.53** | - | 0.29** | 0.35** | 0.40** | 0.51** | -0.15** | -0.18** | -0.21** | -0.18** |
| 1. Externalizing problems, age 3 | 0.38** | 0.30** | 0.31** | 0.28** | - | 0.60** | 0.54** | 0.48** | -0.27** | -0.23** | -0.26** | -0.20** |
| 1. Externalizing problems, age 5 | 0.27** | 0.40** | 0.35** | 0.32** | 0.60** | - | 0.70** | 0.61** | -0.24** | -0.24** | -0.28** | -0.21** |
| 1. Externalizing problems, age 7 | 0.25** | 0.31** | 0.43** | 0.35** | 0.53** | 0.69** | - | 0.69** | -0.21** | -0.23** | -0.31** | -0.22** |
| 1. Externalizing problems, age 11 | 0.23** | 0.27** | 0.34** | 0.49** | 0.47** | 0.58** | 0.67** | - | -0.21** | -0.23** | -0.30** | -0.25** |
| 1. IQ, age 3 | -0.23** | -0.21** | -0.19** | -0.19** | -0.27** | -0.26** | -0.25** | -0.24** | - | 0.52** | 0.48** | 0.34** |
| 1. IQ, age 5 | -0.17** | -0.20** | -0.18** | -0.17** | -0.21** | -0.25** | -0.23** | -0.22** | 0.52** | - | 0.61** | 0.41** |
| 1. IQ, age 7 | -0.18** | -0.20** | -0.22** | -0.24** | -0.24** | -0.29** | -0.31** | -0.30** | 0.46** | 0.60** | - | 0.41** |
| 1. IQ, age 11 | -0.18** | -0.16** | -0.17** | -0.19** | -0.19** | -0.22** | -0.21** | -0.23** | 0.35** | 0.39** | 0.40** | - |
| Males: N | 7,829 | 7,455 | 6,791 | 6,413 | 7,306 | 7,437 | 6,791 | 6,406 | 6,706 | 7,543 | 6,654 | 6,506 |
| Mean | 3.02 | 2.60 | 2.83 | 3.24 | 7.14 | 5.31 | 5.35 | 5.08 | 98.15 | 98.92 | 99.53 | 100.61 |
| SD | 2.57 | 2.61 | 2.93 | 3.23 | 3.88 | 3.55 | 3.75 | 3.78 | 15.08 | 15.35 | 15.66 | 15.30 |
| Females: N | 7,084 | 7,147 | 6,570 | 6,318 | 7,074 | 7,140 | 6,569 | 6,303 | 6,748 | 7,249 | 6,567 | 6,431 |
| Mean | 2.81 | 2.50 | 2.68 | 3.21 | 6.30 | 4.31 | 4.14 | 3.89 | 102.05 | 101.28 | 100.59 | 99.48 |
| SD | 2.48 | 2.46 | 2.66 | 3.09 | 3.70 | 3.21 | 3.32 | 3.27 | 14.57 | 14.44 | 14.25 | 14.55 |
| Note: correlations for males/females are presented above/below the diagonal  *p<0.05  **p<0.01 | | | | | | | | | | | | |

| **Table S2.** Fit indices of competing 1- to 6-class parallel process growth mixture models | | | | | | |
| --- | --- | --- | --- | --- | --- | --- |
|  | **1 Class** | **2 Classes** | **3 Classes** | **4 Classes** | **5 Classes** | **6 Classes** |
| **BIC** | 965405.199 | 961902.449 | 959774.984 | 958933.474 | 958360.942 | 956606.683 |
| **AIC** | 965010.880 | 961330.300 | 959063.663 | 958044.323 | 957293.960 | 957851.495 |
| **SSA-BIC** | 965243.124 | 961667.282 | 959482.614 | 958568.012 | 957922.387 | 957339.847 |
| **Entropy** | --- | 0.908 | 0.891 | 0.883 | 0.863 | 0.851 |
| ***Group Sizes*** n (%) |  |  |  |  |  |  |
| Males, Class 1 | 8593 (51%) | 7500 (45%) | 945 (6%) | 573 (3%) | 433 (3%) | 6389 (38%) |
| Males, Class 2 |  | 993 (6%) | 7118 (42%) | 7000 (42%) | 6674 (40%) | 503 (3%) |
| Males, Class 3 |  |  | 530 (3%) | 501 (3%) | 709 (4%) | 516 (3%) |
| Males, Class 4 |  |  |  | 519 (3%) | 568 (3%) | 413 (2%) |
| Males, Class 5 |  |  |  |  | 209 (1%) | 133 (1%) |
| Males, Class 6 |  |  |  |  |  | 639 (4%) |
|  |  |  |  |  |  |  |
| Females, Class 1 | 8251 (49%) | 742 (4%) | 6991 (42%) | 6927 (41%) | 256 (2%) | 262 (2%) |
| Females, Class 2 |  | 7509 (45%) | 620 (4%) | 544 (3%) | 147 (1%) | 159 (1%) |
| Females, Class 3 |  |  | 640 (4%) | 348 (2%) | 653 (4%) | 6379 (38%) |
| Females, Class 4 |  |  |  | 432 (3%) | 759 (5%) | 617 (4%) |
| Females, Class 5 |  |  |  |  | 6436 (38%) | 634 (4%) |
| Females, Class 6 |  |  |  |  |  | 200 (1%) |
| Shaded cells indicate the selected model; BIC=Bayesian information criterion; AIC=Akaike information criterion; SSA-BIC=Sample size adjusted BIC | | | | | | |

| **Table S3.** Crude and adjusted regression estimates (SE) of class membership on decision-making using the CGT stratified by sex | | | | | | | | | |
| --- | --- | --- | --- | --- | --- | --- | --- | --- | --- |
|  | **Risk taking** | | | **Delay aversion** | | | **Deliberation time** | | |
|  | **Crude** | **Model A^a^** | **Model B^b^** | **Crude** | **Model A^a^** | **Model B^b^** | **Crude** | **Model A^a^** | **Model B^b^** |
| **Males** |  |  |  |  |  |  |  |  |  |
| Typically Developing | Ref | Ref | Ref | Ref | Ref | Ref | Ref | Ref | Ref |
| Improvers | **0.03 (0.01)** | 0.02 (0.01) | 0.02 (0.01) | -0.02 (0.02) | -0.03 (0.02) | -0.02 (0.02) | -0.08 (0.08) | -0.04 (0.08) | -0.07 (0.08) |
| Deteriorators | 0.02 (0.01) | 0.02 (0.01) | 0.01 (0.01) | 0.02 (0.02) | 0.02 (0.02) | 0.02 (0.02) | 0.12 (0.08) | 0.11 (0.09) | 0.10 (0.09) |
| Troubled | -0.01 (0.01) | -0.01 (0.01) | -0.01 (0.01) | 0.04 (0.02)* | 0.04 (0.02)* | 0.04 (0.02)* | 0.10 (0.08) | 0.10 (0.08) | 0.07 (0.08) |
| **Females** |  |  |  |  |  |  |  |  |  |
| Typically Developing | Ref | Ref | Ref | Ref | Ref | Ref | Ref | Ref | Ref |
| Improvers | **0.03 (0.01)** | 0.01 (0.01) | 0.01 (0.01) | 0.02 (0.02) | 0.00 (0.02) | -0.00 (0.02) | 0.01 (0.15) | 0.05 (0.15) | 0.05 (0.15) |
| Deteriorators | **0.04 (0.01)** | **0.04 (0.01)** | **0.04 (0.01)** | **0.06 (0.02)** | **0.05 (0.02)** | 0.05 (0.02)* | 0.31 (0.13)* | 0.30 (0.13)* | 0.31 (0.13)* |
| Troubled | -0.00 (0.01) | -0.01 (0.01) | -0.02 (0.01) | 0.01 (0.03) | 0.01 (0.03) | -0.01 (0.03) | 0.27 (0.12)* | 0.26 (0.13)* | 0.28 (0.13)* |

Bold indicates p<0.01.

*Significant at the 0.05 level

^a^Adjustments for socioeconomic disadvantage, ethnicity, maternal education, birth weight, maternal age at birth, maternal smoking and family structure

^b^Adjustments for Model A covariates + maternal psychological distress, parent-child relationship, harsh parental discipline, household chaos, quality of emotional support, parental involvement, regular bedtimes and breastfeeding

| **Table S4.** Crude and adjusted regression estimates (SE) of class membership on decision-making using the CGT stratified by sex (continued) | | | | | | | | | |
| --- | --- | --- | --- | --- | --- | --- | --- | --- | --- |
|  | **Overall proportion bet** | | | **Quality of decision-making** | | | **Risk adjustment** | | |
|  | **Crude** | **Model A^a^** | **Model B^b^** | **Crude** | **Model A^a^** | **Model B^b^** | **Crude** | **Model A^a^** | **Model B^b^** |
| **Males** |  |  |  |  |  |  |  |  |  |
| Typically Developing | Ref | Ref | Ref | Ref | Ref | Ref | Ref | Ref | Ref |
| Improvers | **0.03 (0.01)** | 0.02 (0.01)* | 0.02 (0.01) | 0.01 (0.01) | 0.02 (0.01) | 0.02 (0.01) | -0.01 (0.05) | 0.07 (0.06) | 0.08 (0.06) |
| Deteriorators | 0.02 (0.01)* | 0.02 (0.01)* | 0.02 (0.01) | -0.02 (0.01)* | -0.01 (0.01) | -0.01 (0.01) | -0.16 (0.06)* | -0.12 (0.07) | -0.11 (0.07) |
| Troubled | 0.01 (0.01) | 0.00 (0.01) | 0.00 (0.01) | **-0.03 (0.01)** | -0.03 (0.01)* | -0.02 (0.01)* | **-0.19 (0.07)** | -0.14 (0.07)* | -0.14 (0.07)* |
| **Females** |  |  |  |  |  |  |  |  |  |
| Typically Developing | Ref | Ref | Ref | Ref | Ref | Ref | Ref | Ref | Ref |
| Improvers | **0.04 (0.01)** | 0.02 (0.01) | 0.01 (0.01) | -0.02 (0.01) | -0.02 (0.01) | -0.02 (0.01) | -0.13 (0.06)* | -0.05 (0.06) | -0.03 (0.06) |
| Deteriorators | **0.04 (0.01)** | **0.04 (0.01)** | **0.03 (0.01)** | **-0.04 (0.01)** | -0.03 (0.01)* | -0.03 (0.01)* | **-0.27 (0.07)** | **-0.23 (0.07)** | **-0.22 (0.07)** |
| Troubled | 0.00 (0.01) | -0.00 (0.01) | -0.01 (0.01) | -0.02 (0.01) | -0.01 (0.01) | -0.01 (0.02) | **-0.28 (0.07)** | **-0.19 (0.07)** | -0.16 (0.08)* |

Bold indicates p<0.01.

*Significant at the 0.05 level

^a^Adjustments for socioeconomic disadvantage, ethnicity, maternal education, birth weight, maternal age at birth, maternal smoking and family structure

^b^Adjustments for Model A covariates + maternal psychological distress, parent-child relationship, harsh parental discipline, household chaos, quality of emotional support, parental involvement, regular bedtimes and breastfeeding

| **Table S5.** Crude and adjusted OR (95%CI) of multinomial logit regression models examining the relationship between class membership and bullying involvement stratified by sex | | | | | | | | | | | | |
| --- | --- | --- | --- | --- | --- | --- | --- | --- | --- | --- | --- | --- |
|  | **Crude** | | | | **Model A^a^** | | | | **Model B^b^** | | | |
|  | **Neutral** | **Bully** | **Bully-**  **victim** | **Victim** | **Neutral** | **Bully** | **Bully-**  **victim** | **Victim** | **Neutral** | **Bully** | **Bully-**  **victim** | **Victim** |
| **Males** |  |  |  |  |  |  |  |  |  |  |  |  |
| Typically Developing | Ref | Ref | Ref | Ref | Ref | Ref | Ref | Ref | Ref | Ref | Ref | Ref |
| Improvers | Ref | 0.93  (0.46-1.90) | 0.90  (0.67-1.22) | 0.89  (0.64-1.24) | Ref | 0.73  (0.35-1.53) | 0.94  (0.69-1.27) | 1.00  (0.72-1.40) | Ref | 0.68  (0.32-1.46) | 0.87  (0.64-1.18) | 0.94  (0.67-1.33) |
| Deteriorators | Ref | **2.07**  **(1.20-3.57)** | **3.06**  **(2.26-4.14)** | **2.23**  **(1.61-3.08)** | Ref | **1.93**  **(1.11-3.36)** | **3.04**  **(2.24-4.12)** | **2.20**  **(1.59-3.04)** | Ref | 1.80  (1.04-3.11)* | **2.98**  **(2.19-4.06)** | **2.16**  **(1.56-2.99)** |
| Troubled | Ref | 1.02  (0.57-1.80) | **2.27**  **(1.64-3.13)** | **2.47**  **(1.75-3.49)** | Ref | 0.89  (0.50-1.58) | **2.25**  **(1.64-3.11)** | **2.49**  **(1.78-3.50)** | Ref | 0.87  (0.48-1.57) | **2.16**  **(1.57-2.98)** | **2.38**  **(1.68-3.37)** |
| **Females** |  |  |  |  |  |  |  |  |  |  |  |  |
| Typically Developing | Ref | Ref | Ref | Ref | Ref | Ref | Ref | Ref | Ref | Ref | Ref | Ref |
| Improvers | Ref | 1.80  (0.74-4.44) | 1.01  (0.72-1.41) | 0.86  (0.82-1.42) | Ref | 1.31  (0.48-3.56) | 0.92  (0.64-1.31) | 1.12  (0.83-1.49) | Ref | 1.23  (0.43-3.54) | 0.87  (0.62-1.23) | 1.09  (0.82-1.46) |
| Deteriorators | Ref | 1.31  (0.55-3.10) | **2.49**  **(1.73-3.57)** | **2.81**  **(2.06-3.83)** | Ref | 1.20  (0.49-2.93) | **2.39**  **(1.66-3.45)** | **2.77**  **(2.01-3.81)** | Ref | 1.11  (0.44-2.81) | **2.34**  **(1.62-3.37)** | **2.74**  **(1.99-3.78)** |
| Troubled | Ref | 1.90  (0.61-5.90) | **2.11**  **(1.34-3.32)** | **2.91**  **(2.06-4.12)** | Ref | 1.50  (0.47-4.74) | **1.95**  **(1.34-3.08)** | **2.82**  **(1.98-4.02)** | Ref | 1.45  (0.46-4.50) | 1.76  (1.11-2.79)* | **2.70**  **(1.90-3.84)** |

Bold indicates p<0.01.

*Significant at the 0.05 level

^a^Adjustments for socioeconomic disadvantage, ethnicity, maternal education, birth weight, maternal age at birth, maternal smoking and family structure

^b^Adjustments for Model A covariates + maternal psychological distress, parent-child relationship, harsh parental discipline, household chaos, quality of emotional support, parental involvement, regular bedtimes and breastfeeding

| **Table S6.** Crude and adjusted OR (95%CI) of logistic and ordered logistic regression models examining the relationship of class membership with truancy, school liking and happiness stratified by sex | | | | | | | | | |
| --- | --- | --- | --- | --- | --- | --- | --- | --- | --- |
|  | **Truancy** | | | **School (dis)liking** | | | **(Un)happiness** | | |
|  | **Crude** | **Model A^a^** | **Model B^b^** | **Crude** | **Model A^a^** | **Model B^b^** | **Crude** | **Model A^a^** | **Model B^b^** |
| **Males** |  |  |  |  |  |  |  |  |  |
| Typically Developing | Ref | Ref | Ref | Ref | Ref | Ref | Ref | Ref | Ref |
| Improvers | **2.01**  **(1.24-3.28)** | 1.44  (0.85-2.42) | 1.25  (0.72-2.18) | 1.00  (0.79-1.26) | 1.12  (0.89-1.40) | 1.06  (0.84-1.33) | 0.98  (0.73-1.31) | 1.00 (0.74-1.35) | 0.88  (0.64-1.21) |
| Deteriorators | **3.71**  **(2.61-5.28)** | **3.01**  **(2.10-4.32)** | **2.83**  **(1.95-4.11)** | **2.04**  **(1.60-2.62)** | **1.82**  **(1.41-2.36)** | **1.75**  **(1.34-2.27)** | **3.31**  **(2.53-4.33)** | **3.11**  **(2.37-4.09)** | **2.97**  **(2.25-3.92)** |
| Troubled | 1.41  (0.78-2.55) | 1.13  (0.62-2.04) | 1.06  (0.56-1.98) | **1.68**  **(1.27-2.21)** | **1.59**  **(1.20-2.12)** | **1.54**  **(1.16-2.05)** | **2.92**  **(2.17-3.92)** | **2.79**  **(2.06-3.77)** | **2.56**  **(1.87-3.50)** |
| **Females** |  |  |  |  |  |  |  |  |  |
| Typically Developing | Ref | Ref | Ref | Ref | Ref | Ref | Ref | Ref | Ref |
| Improvers | **3.09**  **(1.77-5.40)** | **2.34**  **(1.24-4.40)** | **2.35**  **(1.24-4.43)** | 1.01  (0.76-1.35) | 1.11  (0.84-1.49 | 1.07  (0.80-1.43) | **1.54**  **(1.14-2.09)** | **1.60**  **(1.17-2.19)** | 1.47  (1.06-2.06)* |
| Deteriorators | **2.51**  **(1.28-4.92)** | 2.11  (1.11-4.00)* | 2.22  (1.17-4.22)* | **2.05**  **(1.59-2.64)** | **1.97**  **(1.52-2.55)** | **1.92**  **(1.48-2.50)** | **3.06**  **(2.38-3.93)** | **2.86**  **(2.20-3.71)** | **2.76**  **(2.12-3.59)** |
| Troubled | **5.31**  **(2.96-9.53)** | **3.89**  **(2.13-7.10)** | **4.15**  **(2.14-8.04)** | **1.77**  **(1.30-2.41)** | **1.64**  **(1.18-2.27)** | 1.51  (1.08-2.10)* | **2.59**  **(1.83-3.66)** | **2.27**  **(1.59-3.24)** | **1.90**  **(1.30-2.78)** |

Bold indicates p<0.01.

*Significant at the 0.05 level

^a^Adjustments for socioeconomic disadvantage, ethnicity, maternal education, birth weight, maternal age at birth, maternal smoking and family structure

^b^Adjustments for Model A covariates + maternal psychological distress, parent-child relationship, harsh parental discipline, household chaos, quality of emotional support, parental involvement, regular bedtimes and breastfeeding

| **Table S7.** Crude and adjusted OR (95%CI) of logistic regression models examining the relationship of class membership with antisocial behavior and smoking/drinking status stratified by sex | | | | | | |
| --- | --- | --- | --- | --- | --- | --- |
|  | **Antisocial behavior** | | | **Smoking/drinking** | | |
|  | **Crude** | **Model A^a^** | **Model B^b^** | **Crude** | **Model A^a^** | **Model B^b^** |
| **Males** |  |  |  |  |  |  |
| Typically Developing | Ref | Ref | Ref | Ref | Ref | Ref |
| Improvers | 0.84 (0.62-1.14) | 0.74 (0.54-1.01) | 0.66 (0.48-0.91)* | 0.77 (0.53-1.11) | 0.85 (0.59-1.22) | 0.75 (0.52-1.10) |
| Deteriorators | **2.37 (1.89-2.98)** | **2.06 (1.64-2.60)** | **1.94 (1.54-2.44)** | **1.99 (1.45-2.72)** | **1.70 (1.23-2.35)** | **1.59 (1.15-2.20)** |
| Troubled | **1.87 (1.43-2.43)** | **1.65 (1.25-2.18)** | **1.52 (1.14-2.02)** | 1.07 (0.70-1.64) | 0.95 (0.62-1.46) | 0.87 (0.57-1.34) |
| **Females** |  |  |  |  |  |  |
| Typically Developing | Ref | Ref | Ref | Ref | Ref | Ref |
| Improvers | **1.57 (1.16-2.12)** | 1.39 (1.01-1.92)* | 1.26 (0.92-1.74) | 1.20 (0.76-1.88) | 1.42 (0.90-2.23) | 1.28 (0.82-1.99) |
| Deteriorators | **2.22 (1.66-2.96)** | **2.03 (1.50-2.74)** | **1.95 (1.44-2.65)** | 1.40 (0.86-2.28) | 1.25 (0.77-2.04) | 1.21 (0.75-1.96) |
| Troubled | **1.95 (1.38-2.76)** | 1.56 (1.11-2.19)* | 1.31 (0.91-1.87) | 1.01 (0.60-1.69) | 0.83 (0.49-1.43) | 0.67 (0.38-1.18) |

Bold indicates p<0.01.

*Significant at the 0.05 level

^a^Adjustments for socioeconomic disadvantage, ethnicity, maternal education, birth weight, maternal age at birth, maternal smoking and family structure

^b^Adjustments for Model A covariates + maternal psychological distress, parent-child relationship, harsh parental discipline, household chaos, quality of emotional support, parental involvement, regular bedtimes and breastfeeding

| **Table S8.** Crude and adjusted regression estimates (SE) of class membership on self-esteem stratified by sex | | | |
| --- | --- | --- | --- |
|  | **(Low) self-esteem** | | |
|  | **Crude** | **Model A^a^** | **Model B^b^** |
| **Males** |  |  |  |
| Typically Developing | Ref | Ref | Ref |
| Improvers | 0.13 (0.14) | 0.21 (0.14) | 0.15 (0.15) |
| Deteriorators | **0.71 (0.16)** | **0.65 (0.16)** | **0.62 (0.16)** |
| Troubled | **0.76 (0.17)** | **0.74 (0.17)** | **0.71 (0.17)** |
| **Females** |  |  |  |
| Typically Developing | Ref | Ref | Ref |
| Improvers | 0.40 (0.16)* | **0.51 (0.16)** | 0.44 (0.17)* |
| Deteriorators | **0.81 (0.17)** | **0.75 (0.17)** | **0.71 (0.17)** |
| Troubled | **0.77 (0.21)** | **0.67 (0.20)** | **0.54 (0.20)** |

Bold indicates p<0.01.

*Significant at the 0.05 level

^a^Adjustments for socioeconomic disadvantage, ethnicity, maternal education, birth weight, maternal age at birth, maternal smoking and family structure

^b^Adjustments for Model A covariates + maternal psychological distress, parent-child relationship, harsh parental discipline, household chaos, quality of emotional support, parental involvement, regular bedtimes and breastfeeding
